# Pictorial review of MRI/CT Scan in congenital temporal bone anomalies, in patients for cochlear implant

**DOI:** 10.4103/0971-3026.50825

**Published:** 2009-05

**Authors:** Santosh S Gupta, Shailendra R Maheshwari, Milind V Kirtane, Nitin Shrivastav

**Affiliations:** Department of Radiology, P.D. Hinduja National Hospital and Medical Research Centre, Mumbai, India; 1 Department of ENT, P.D. Hinduja National Hospital and Medical Research Centre, Mumbai, India

**Keywords:** Cochlear implant, HRCT, MRI, temporal bone

## Abstract

High-resolution CT scan (HRCT) and MRI are routinely performed prior to cochlear implant surgery. These modalities help assess the status of the inner ear structures. A few patients have significant anomalies, which need to be assessed and understood in detail. We present a pictorial essay of these anomalies and described our HRCT and MRI techniques in patients being imaged prior to surgery.

## Introduction

Over the last decade there has been tremendous growth in the number of cochlear implants being performed and, consequently, there has also been a steady increase in the imaging, done as a part of the preoperative workup of these patients. High-resolution computed tomography (HRCT) and MRI of the temporal bones provide vital information; these are baseline investigations and are necessary in all patients posted for cochlear implant surgery. MRI is now increasingly being used to study the membranous labyrinth and the cranial nerves; it provides accurate information and exquisite anatomical detail.[1]

This paper is a pictorial essay on the various congenital temporal bone anomalies seen in patients being investigated prior to cochlear implant surgery. There are several complex congenital anomalies that are encountered while imaging such patients. The radiologist needs to follow a clinically oriented classification of these anomalies, which helps the implant surgeon plan the correct management strategy. A classification widely used by otolaryngologists is the one described by Sennaroglu and Saatci,[2] and we too have used this system with a few modifications.

## CT and MRI Techniques

### HRCT

HRCT scans are performed on a 64-slice volume scanner (LightSpeed VCT, GE, Milwaukee, USA) in a straight axial plane (kV: 140, mA: 350, matrix: 512 × 512, slice thickness: 0.625 mm/10.63, 0.531:1, scan field of view (FOV): 32 cm, display FOV: 9.6 cm). The original isometric volume data is used to obtain coronal reformatted images. The images are reviewed with a high-resolution bone algorithm, using a small FOV for separate right and left ear documentation.

### MRI

**Figure 1 (A-C):**
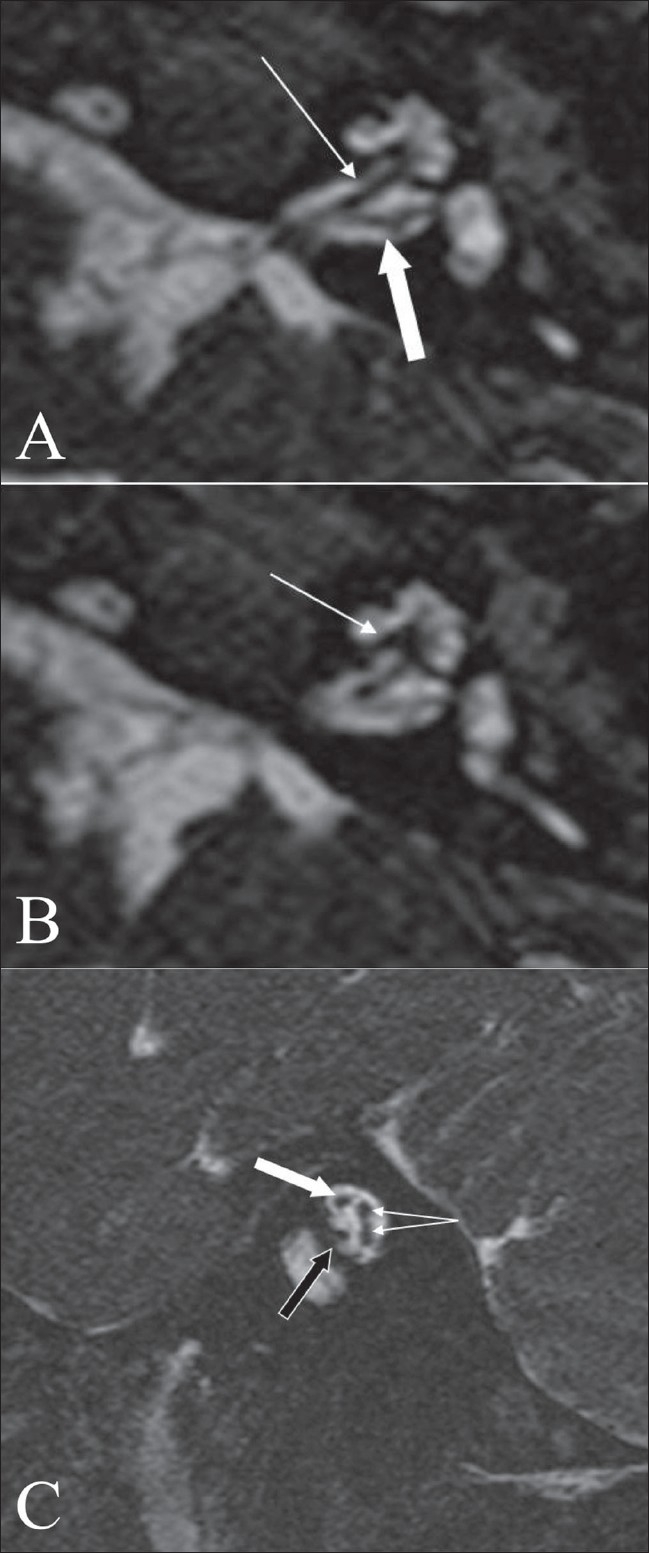
Normal anatomy. An axial 3D-FIESTA image (A), at the level of the vestibule and cochlea shows the cochlear (arrow) and vestibular (block arrow) nerves. An axial 3D-FIESTA image (B), at the level of the vestibule and cochlea, shows a well-delineated and intact modiolus (arrow). An oblique sagittal 3D FIESTA image (C) through the internal auditory canal shows the facial nerve in the antero-superior quadrant (block white arrow), the cochlear nerve in the antero-inferior quadrant (block black arrow) and the superior and inferior vestibular nerves, in the posterior quadrant (branching arrows)

MRI scans are performed on 1.5-T MR machine (Excite Twin Speed, GE, Milwaukee, USA) with an 8-channel head coil. Sedation is used in most patients. A 3D-FIESTA (fast imaging enabling steady-state acquisition) axial sequence (TR: 5.5, TE: 1.7/Fr, FOV: 16 × 16, slice thickness: 1.0/−0.5, matrix: 320 × 320, NEX: 6.0) is performed followed by coronal reformations along with 3D maximum intensity projection (MIP) reconstructions. A 3D-FIESTA sequence is also acquired in a direct oblique sagittal plane (TR: 6.7, TE: 2.1/Fr, FOV: 12 × 12, slice thickness: 1.0/−0.5, matrix: 384 × 320, NEX: 6.0) perpendicular to the VII–VIII nerve complexes. With this technique we are able to obtain better resolution than with reformations from an axial sequence; this enables better delineation of the nerves [Figure 1]. A routine T2W axial sequence through the brain is obtained in all patients.

## Discussion

Congenital malformations of the inner ear are rare anomalies; they can be identified on imaging with HRCT and/or MRI in about 20% of patients with congenital sensorineural hearing loss.[3]

**Figure 2 (A-D):**
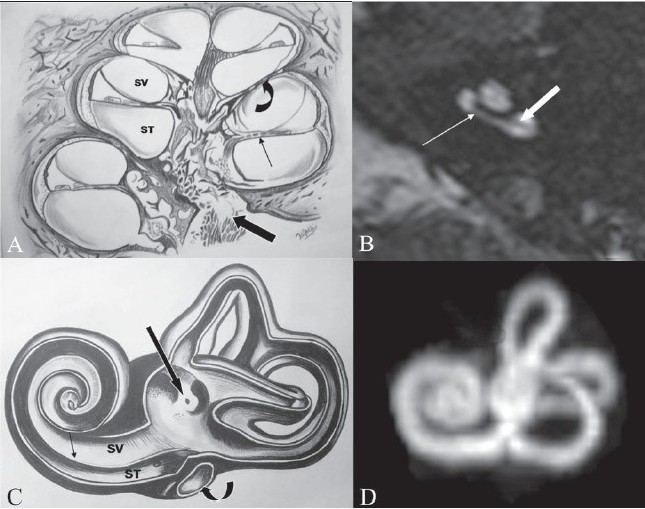
Normal anatomy. Line diagram (A) of the internal structure of the cochlea shows the modiolus (block arrow), interscalar septum (curved arrow) and the osseous spiral lamina (thin arrow), the latter separating the scala vestibuli (SV) from the scala tymapni (ST). An axial 3D-FIESTA image (B), at the level of cochlea shows the interscalar septum (arrow) and the osseous spiral lamina (block arrow). A line diagram (C) shows the internal architecture of the membranous labyrinth, with the osseous spiral lamina (arrow) separating the SV from the ST. Note the vestibule, with the orifice for the vestibular aqueduct (block black arrow). The curved arrow shows the fenestra cochlea. A 3D-MIP reconstruction (D) of the data from the 3D FIESTA sequence shows the anatomy of the membranous labyrinth

Based on the site of abnormality, congenital inner ear anomalies can be classified into:

(a) cochlear malformations, (b) vestibular malformations, (c) malformations of the semicircular canals, (d) vestibular and cochlear aqueduct malformations, (e) cochlear nerve deficiency, (f) isolated attenuated modiolus, and (g) isolated cochlea. A classification commonly used by ENT surgeons is the one described by Sennaroglu and Saatci[2]; we have used this system with some modifications since we encountered some additional anomalies on MRI (such as cochlear nerve deficiency) With the resolution provided by the newer CT scan and MRI equipments, it is now possible to see minute internal structures of the cochlea such as the interscalar septum [Figure 2a] which divides the major cochlear turns; this can be seen on both HRCT and MRI 3D-FIESTA sequences [Figure 2b]. The osseous spiral lamina, a thin membrane within each turn of the cochlea that separates the scala vestibuli from the scala tympani,[4] can also be well seen on MRI as a thin, linear structure [Figure 2b]. The MRI 3D sequence data can be used to obtain a 3D MIP reconstruction [Figure 2d], which gives a good outline of the inner ear structures, especially when complex anomalies need evaluation. The membranous labyrinth contains endolymph and is surrounded by the perilymph which, in turn, separates it from the otic capsule or bony labyrinth. The cochlea consists of two and one half turns, which extend into the vestibule. The three semicircular canals arise from the vestibule in arches along all three planes. Laterally the otic capsule is called the promontory and is thickest over the basal turn of the cochlea; posteriorly, it is perforated by the round and oval windows [Figure 2c].

The vestibular aqueduct represents an osseous aperture in the bony labyrinth; it is about 5 mm in length and is located along the medial aspect of the pyramid. Although its lumen is lined by squamous and cuboidal epithelium, it houses an extension of the membranous labyrinth—the endolymphatic duct. On axial CT scan, the vestibular aqueduct is seen as a small slit running medial and parallel to the plane of the posterior semicircular canal. Its distal, external funnel-like opening, much like the external opening of the cochlear aqueduct, can usually be visualized on a CT scan, when it can be seen opening into a linear ridge of bone—the foveate impression.[5]

The seventh–eighth nerve complexes are well seen on MRI. On the 3D-FIESTA sequence, it is possible to identify further divisions of the eighth nerve [Figure 1a]. The cochlear nerve is well seen on axial 3D-FIESTA images [Figure 1b], extending into the modiolus. The anatomy and delineation of the nerves is better appreciated in an oblique sagittal plane, perpendicular to the plane of the internal auditory canal.[6] With this technique [Figure 1c], the superior and inferior vestibular nerves are seen in the posterior quadrants and the cochlear nerve in the anteroinferior quadrant, while the facial nerve is seen in the anterosuperior quadrant.

The important congenital anomalies that are encountered when imaging patients prior to cochlear implant surgeries are discussed in detail below. Some of the malformations, such as those of the semicircular canals, have not been discussed, since they do not impact the surgery or management.

### Michel deformity:

**Figure 3 (A-E):**
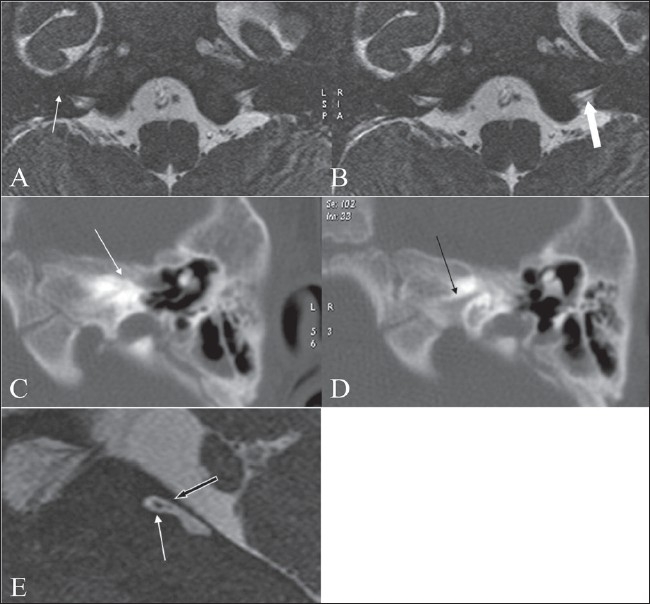
Michel deformity. Axial 3D-FIESTA images (A, B) show absence of the entire vestibulo- cochlear structures bilaterally (arrowd). The internal auditory canals are small on both sides, with a markedly thin eighth nerve (white arrow in B). HRCT images (C, D) shows a small left internal auditory canal (black arrow in D) and absence of the entire vestibulo- cochlear apparatus (white arrow in C), consistent with a Michel deformity. An oblique sagittal 3D FIESTA image (E), through the small internal auditory canal shows a thinned-out eighth nerve (block back arrow) with no divisions and a normal facial nerve antero-superiorly (arrow)

In this deformity, there is absence of the entire cochlea and the vestibular structures, i.e., complete labyrinthine aplasia[2] It may be bilateral or unilateral [Figure 3]. The internal auditory canals (IACs) are small in size on both sides [Figure 3a and b]. Cochlear nerve deficiency will be seen [Figure 3E], on MR 3D-FIESTA images.

### Cochlear aplasia:

**Figure 4 (A-H):**
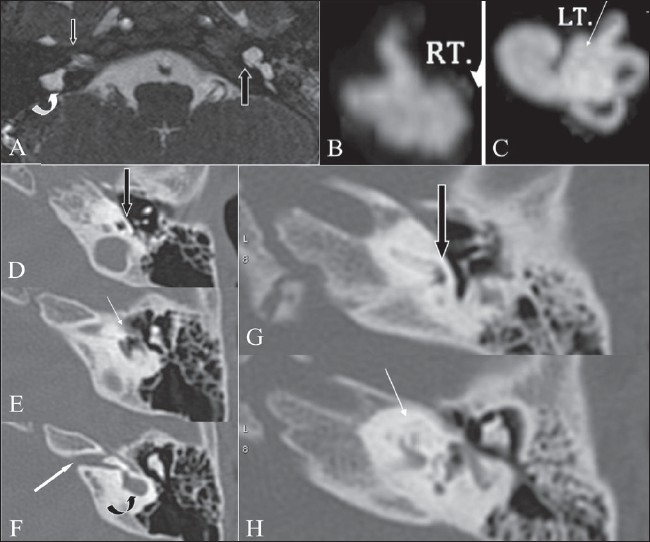
Cochlear aplasia and Incomplete partition type I deformity. Axial 3D-FIESTA image (A) shows an absent right cochlea, suggesting cochlear aplasia (arrow), along with a deformed / dysplastic vestibule (curved arrow). On the left side, a dilated cystic cochlea is noted, with complete lack of the modiolus. 3D-MIP reconstruction images (B, C) of the image data set of Figure 4A, clearly show an absent cochlea on the right (B). On the left (C), a dilated cochlea is noted, with a cystic vestibule, consistent with an incomplete partition type I deformity. Axial HRCT images (D-F) at three consecutive levels show cochlear aplasia (white arrow in E), with loss of the cochlear promontory bulge (block black arrow in D) normally produced by the basal turn of cochlea (compare with Figs. 4G and 4H). At a higher level (F), a small IAC is noted (white arrow) along with a dysplastic cystic vestibule (curved arrow). Axial HRCT images (G, H) of another patient with labyrinthitis ossificans (taken for comparison with Figs 4D, 4E and 4F), show complete ossification of the cochlea (white arrow in H). Note that the normal cochlear promontory bulge (block arrow in G) is maintained

In this condition, the cochlea is completely absent[2] [Figure 4]. The vestibule and semicircular canals may be normal, dilated [Figure 4a, 4b], or hypoplastic. Dense otic bone is present at the site of the cochlea. The appearance may simulate complete labyrinthitis ossificans, in which normal-sized bone is seen anterior to the IAC as also the bulge of the cochlear promontory produced by the basal turn of cochlea; both features are absent in cochlear aplasia [Figure 4d].

### Common cavity deformity:

**Figure 5:**
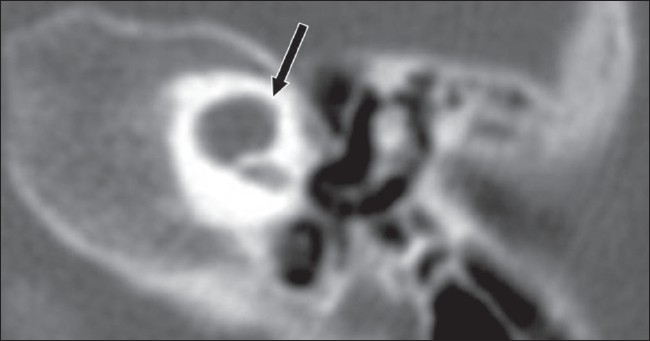
Common cavity. Axial HRCT image shows a common cavity (arrow), in which there is no differentiation between the cochlea and vestibule with a resultant cystic cavity

In this condition there is no differentiation between the cochlea and the vestibule, both together forming a cystic cavity[2] [Figure 5]. This occurs due to developmental arrest in the fourth week of gestation, when differentiation into the cochlea and vestibule has not yet taken place.

### Incomplete partition type I (IP–I):

**Figure 6 (A-E):**
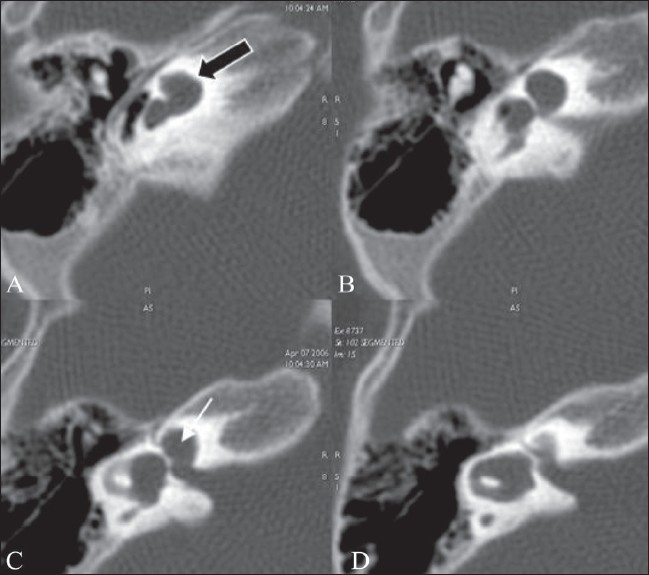
Incomplete partition type I. Successive axial HRCT scans show findings of an incomplete partition type I deformity, with a cystic appearance of the cochlea, lacking the entire modiolus (block black arrow in A) and a cystic vestibule (white arrow in C)

In this condition, the cochlea lacks the entire modiolus and the cribriform area and appears cystic, along with a large cystic vestibule[2] [Figure 6]. The dimensions of the cochlea and the vestibule are normal but the internal architecture, including the modiolus, is missing, giving it an empty cyst-like appearance. The modiolus is completely absent in its entire length from the base to the apex. One needs to note the striking difference from the IP-II (Mondini) deformity, where only the middle and the apical turns form a cystic cavity due to fusion. This pathology represents a form of common cavity that is one step more organized and differentiated than the previously described common cavity malformation, the developmental arrest occurring in the fifth week.[2]

### Cochleo-vestibular hypoplasia:

In this group, the cochlear and the vestibular structures are separate from each other and more differentiated than in IP-II, with failure of development occurring in the sixth week.[2] Both the cochlea and the vestibule are small in size, with the hypoplastic cochlea seen as a small bud, coming off the IAC.[2]

### Incomplete partition type II (IP-II) (Mondini deformity):

**Figure 7 (A-D):**
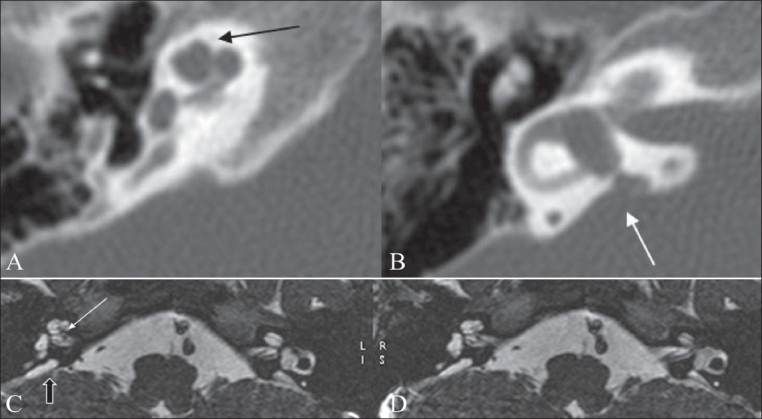
Incomplete partition type II. Axial HRCT images (A, B) show fusion of the middle and apical turns of the cochlea (black arrow in A) and a prominent vestibule and dilated vestibular aqueduct (arrow in B), suggesting an incomplete partition type II (Mondini) deformity. Axial 3D-FIESTA images (C, D) show a dilated vestibular sac (block arrow in C) and a markedly attenuated modiolus (arrow in C)

This condition represents developmental arrest occurring at a later stage than in IP-I (seventh week of gestation), with the size of the cochlea and vestibule appearing normal and internal organization being more developed.[2] The cochlea consists of 1.5 turns, with the middle and apical turns coalescing to form a cystic apex (due to a defect in the interscalar septum), along with a dilated vestibule and enlarged vestibular aqueduct [Figure 7]. The modiolus is present in the basal turn where the ganglion cells and nerve endings are usually seen[2,7] and, therefore, these patients are more likely to regain hearing after cochlear implantation than in patients with IP-I.

### Dilated vestibular aqueduct:

**Figure 8:**
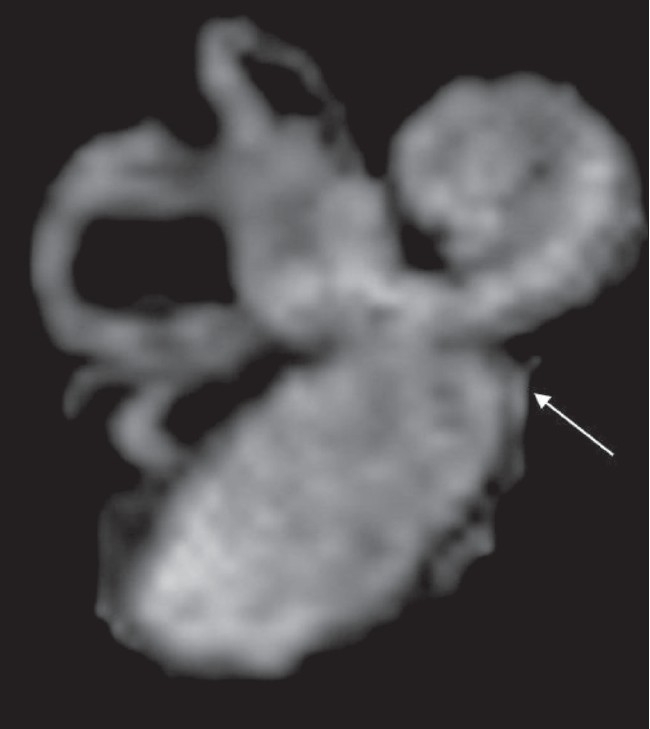
Enlarged vestibular sac. 3D-MIP reconstruction of the images of another patient with IP II demonstrates a large vestibular sac (arrow)

This may occur in isolation [Figure 8] or in combination with other inner ear malformations. However, Lemmerling *et al,*[8] in their series found that all ears with large vestibular aqueducts have an associated modiolar deficiency. Hence the term, isolated dilated vestibular aqueduct seems obsolete. IP-II (classic Mondini) is always associated with a dilated vestibular aqueduct. An isolated dilated vestibular aqueduct has also been observed and reported by other authors.[9] Valvassori *et al*.[10], who were the first to describe an association between an enlarged vestibular aqueduct and sensorineural hearing loss, coined the term ‘large vestibular aqueduct syndrome.’ Interestingly, this may be a part of a syndrome, such as Pendred syndrome and distal renal tubular acidosis,[11] and one needs to look for this possibility in the appropriate setting. Bamiou *et al*, in their series of patients with sensorineural hearing loss evaluated with HRCT, found that 60% of their patients had an isolated dilated vestibular aqueduct.[12] The criterion that they used for a dilated vestibular aqueduct was a middle-third diameter of the duct of more than 1.5 mm.[12]

### Cochlear nerve deficiency:

**Figure 9 (A-D):**
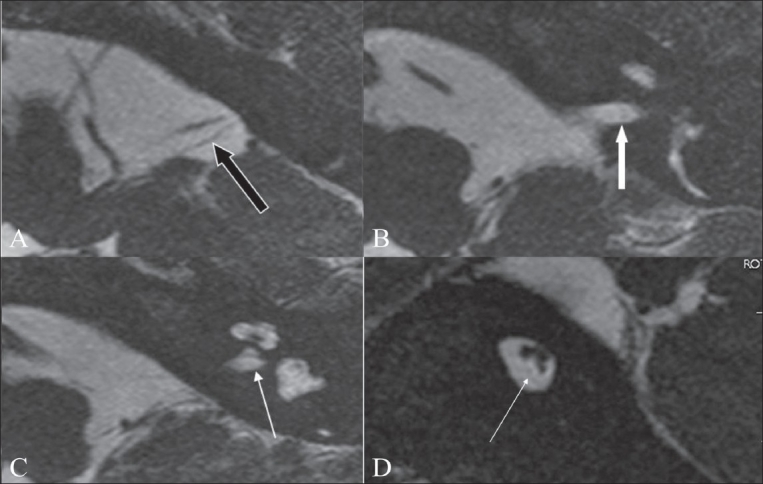
Cochlear nerve deficiency. Axial 3D-FIESTA images (A-C) show a markedly thinned-out eighth nerve in the CP angle cistern (black arrow in A), which is barely seen in the distal portion of the canal (white arrow in B). The left cochlear nerve is not visualized in its normal location (arrow in C). An oblique sagittal 3D-FIESTA image (D) shows the facial and vestibular nerves in their normal location, with the cochlear nerve barely or almost not seen in the antero-inferior quadrant (arrow)

We have used the term cochlear nerve deficiency to encompass both absent [Figure 3 and 9] as well as hypoplastic cochlear nerves, based on the study by Glastonbury *et al*.[13] This study and another by Kim *et al.*[6] showed that the cochlear nerve is larger than either the superior or inferior vestibular nerves in 90% of normal cases and it is of almost the same size [Figure 1C] or larger than the facial nerve in 64% of cases. The cochlear nerve size is thought to be correlated with the spiral ganglion cell population and, therefore, determination of the nerve caliber may prove to be helpful in predicting the outcome of cochlear implantation.[14] An appreciably thin cochlear nerve, as seen in some of our cases, may still effectively transmit impulses to allow hearing;[15] therefore, MRI depiction of a small nerve is only a relative contraindication to cochlear implantation.

In almost all patients with congenital cochlear nerve deficiency, the IACs are small in size. As per the criteria described in literature, an abnormal IAC is diagnosed if it is <4 mm in either the vertical or transverse diameters, is irregularly shaped, or is appreciably smaller than the IAC of the contralateral side.[13] In a study by Valvassori *et al.*, the IAC was found to be virtually symmetric in healthy individuals, with a difference of <1 mm in 99% of patients and 1–2 mm in 1% of patients.[16]

### Isolated cochlea:

**Figure 10 (A-D):**
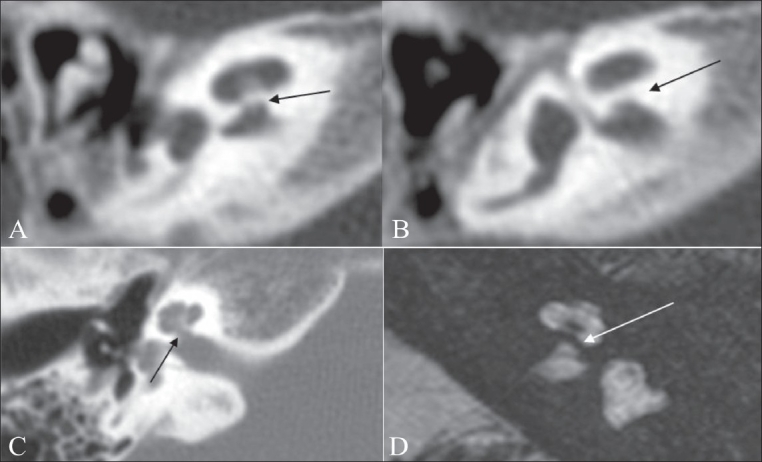
Isolated cochlea. Axial HRCT images (A, B) show a thick bony bar at the cochlear aperture (arrow), suggesting an isolated cochlea. An axial HRCT image (C) shows a normal appearance of the cochlear aperture at the site of the entry of the cochlear nerve, near the modiolus. An axial 3D-FIESTA image (D) shows a bony bar (arrow) at the cochlear aperture (isolated cochlea). Also note the left cochlear nerve deficiency

The term isolated cochlea [Figure 10] is used when the cochlear aperture (which is a small canal at the fundus of the IAC [Figure 10B], through which the cochlear nerve passes to enter the cochlea, is absent and filled with bone.[13] This has also been described as hypoplasia of the bony canal of the cochlear nerve.[17]

### Attenuated modiolus:

This may be an isolated finding or may be seen in association with other malformations. The “modiolus” (taken from the latin word ‘hub of a wheel’) is an irregular conical (or trapezoidal)–shaped porous bone within the cochlea, from which the osseous spiral lamina of the cochlea projects out, which supports the organ of Corti[8] [Figures 1B, 2A].

**Figure 11:**
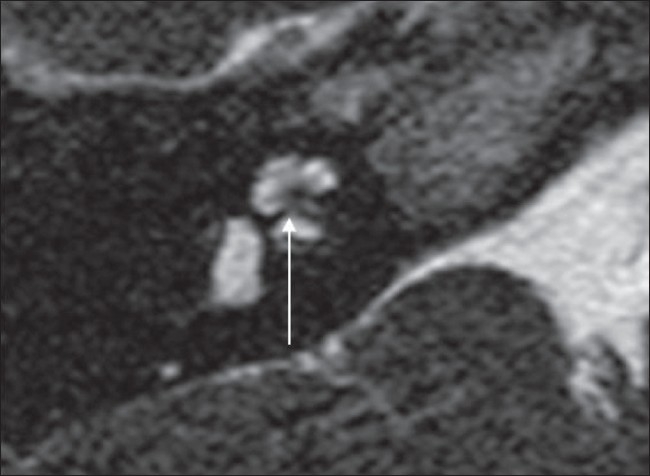
Attenuated modiolus. An axial 3D-FIESTA image shows an attenuated modiolus on the right (arrow). Compare with the normal modiolus in figure 1B

We label a modiolus as attenuated on the basis of subjective criteria [Figure 11], although the more accurate method is the measurement of its area at the point of its maximum size. According to Naganawa *et al*., the size of the modiolus on MRI in normal volunteers is about 4.0 mm^2^.[18]

## Surgical implications for implantation in cochlear malformations

It is important to understand the surgical approach and strategy in patients with cochlear malformations, especially so since there was a time when cochlear implantation was thought to be contraindicated in many of these cases.

Apart from the technical difficulties associated with the surgery, the expectations for improved auditory performance after cochlear implants in patients with inner ear malformations are relatively low; this may be due to a substantially reduced population of spiral ganglion cells[19] and other coexisting abnormalities. It is clear that the approach in each case should be tailored according to the type of malformation. Michel deformity is obviously a contraindication to cochlear implantation, and auditory brainstem implantation may be considered as an option in these cases. In common cavity malformations, the exact location and amount of neural tissue are not definitely known, and the surgeon can use full-banded implants rather than the half-banded ones oriented towards the modiolus. In such cases, as suggested by McElveen *et al*., the use of a precurved electrode may help in avoiding the risk of the electrode entering the IAC.[20] In a hypoplastic cochlea, due to the lack of space, the electrode may enter the IAC if a full insertion is attempted. As reported by Tucci *et al*., the reason for this is that the small space of a hypoplastic cochlea does not allow the electrode to curl within the cavity. In cases of IP-II, the modiolus and the basal turn are present and the surgical approach is similar to the one used in normal cases.[20]

The transmastoid facial recess approach is usually the standard technique for electrode array placement in the normal cochlea; this, however, may not be the best approach for patients with common cavity deformities or cochlear hypoplasia. These patients usually have a thin or absent cribriform area between the IAC and the common cavity and are at high risk for an intraoperative cerebrospinal fluid (CSF) gusher or postoperative CSF leak. An intraoperative CSF leak can be also seen with an IP-II deformity or in the presence of an enlarged IAC.

Cochlear nerve deficiency is not a contraindication for surgery. In those cases where the cochlear nerve is not seen on MRI, it is possible that the nerve is so thin that it is beyond the resolution of a 1.5 T MRI scanner; alternatively, it may be that the cochlear nerve fibers traverse along the vestibular nerve and hence are not detected on MRI. These patients should undergo a hearing aid trial and periodical audiological evaluation by expert audiologists/therapists before a decision is taken on their candidacy for cochlear implant surgery. In all such cases, the side with the more normal-appearing inner ear and with the relatively larger cochlear nerve should be selected for implantation; this is the logical approach. It is also recommended that when the cochlear nerve is not well seen, intracochlear electrical stimulation to determine the auditory nerve action potential and auditory brainstem response may be a valuable test before performing cochlear implantation.[21]

## Conclusion

HRCT and MRI are baseline investigations that need to be done prior to cochlear implant surgery. Both these modalities provide exquisite anatomical details and information. The radiologist should have a clear understanding and knowledge of the various malformations and should follow a simple, clinically oriented, classification that can be easily understood and interpreted by the implant team. There needs to be special emphasis on identifying cochlear malformation and cochlear nerve deficiency since these have a significant impact on cochlear implant surgery and its outcome.
